# Qualitative findings from North America’s first drug compassion club

**DOI:** 10.1371/journal.pone.0315804

**Published:** 2024-12-31

**Authors:** Jeanette Bowles, Eris Nyx, Jeremy Kalicum, Thomas Kerr

**Affiliations:** 1 Department of Medicine, St. Paul’s Hospital, University of British Columbia, Vancouver, BC, Canada; 2 British Columbia Centre on Substance Use, Vancouver, BC, Canada; 3 Drug Users Liberation Front, Canada; Johns Hopkins University Bloomberg School of Public Health, UNITED STATES OF AMERICA

## Abstract

In Canada, the ongoing fatal overdose crisis remains driven by the unpredictable potency and content of the illicit drug supply. From August 2022 until October 2023, the Drug User Liberation Front [DULF] operated a drug compassion club [CC], which sells drugs of known composition and purity without medical oversight. The present study is a qualitative evaluation of this project. From December 2022 to February 2023, we interviewed 16 CC members about their experiences with DULF’s CC. Using a semi-structured interview guide, participants were interviewed in a private space to ensure confidentiality. Thematic analysis was used to code for a priori and unexpected themes. Participants spoke positively of their experiences with the CC, which ranged from lower overdose risk, health improvements, preference for the drug purchasing process, and mutual respect and trust among CC members, founders, and staff. No participants reported overdosing on CC-sourced drugs, and drugs were described as safe and reliable. For opioid users, the tolerance developed for opioid-potent fentanyl hampered the transition to CC heroin. Suggestions for CC improvements were also identified. Despite political backlash to the project, the CC appears to be a novel and promising approach to reducing overdose morbidity in high needs communities. By promoting participant autonomy, regulating an unstable drug supply, and creating community, this intervention has reduced self-reported overdose risk and improved the health and social wellbeing of members. No overdoses reported from CC-sourced drugs suggests that authorizing, expanding and continually evaluating the CC model is warranted.

## Introduction

Each year, fatal overdoses have increased in tandem with the variability in potency of substances and novelty in the unregulated drug supply, this has altered the landscape of drug use in North America [1–3]. In 2023, the overdose fatality rate in Vancouver, British Columbia (B.C.) was 57 per 100,000 people, which in turn has reduced life expectancy in the city [4, 5]. However, in Vancouver’s Downtown Eastside corridor (i.e., “Vancouver–Centre North), the overdose fatality rate was a staggering 560.9 per 100,000 persons [6]; a disparity portraying a snapshot of the area’s milieu as a risk environment in which survival is less likely than other parts of the city [7]. Various medical interventions have been implemented to quell overdose incidents by providing pharmaceutical alternatives to the unregulated drug supply and have been successful insofar as hydromorphone, the primary opioid medication prescribed to offset sole reliance on the unregulated market, was detected in only 3% of overdose fatalities in B.C. in 2023 [8]. Nonetheless, their application through clinician prescribing often requires medical oversight, which might include prescriber determination of medication dose, pickup schedule, daily witnessed consumption requirements, and urinalysis [9–12]. These factors create barriers to engagement for large numbers of individuals at heightened risk of overdose [13]. To address these issues, there have been growing calls for the implementation of drug-user led drug Compassion Clubs (CCs) [14], which are borne from Cannabis Compassion Clubs that operated outside of the law and provided medical cannabis to treat pain, including pain for patients who faced HIV-associated stigma. Cannabis compassion clubs were also found to facilitate social bonds and support [15]. The CC being examined in the present study is an overdose prevention approach that operates outside of the medical system and involves selling drugs of known composition and purity at cost, with the goal of undercutting the illicit market and providing a secure space in which to purchase and use substances [16]. In Vancouver, the first known CC was implemented by the Drug Users Liberation Front [DULF] in the Downtown Eastside and was operational for one-year and two-months. Findings from a quantitative evaluation of the CC revealed reduced risk of fatal and non-fatal overdose occurrence [17]. Although the drugs that were sold were acquired from an unknown origin and purchased from the “Dark Web”; they had a predictable content as they were tested for quality assurance. DULF’s CC operated at increasing capacity beginning with a small group of twenty-four people and expanded as time and resources permitted until a law enforcement raid forced its closure. In the present study, we aimed to qualitatively evaluate the CC, its uptake, processes, and whether it has succeeded in upholding its aims to reduce overdose risk and improve health among members of North America’s first CC.

## Methods & materials

The following study is a qualitative evaluation of the CC following the first 6-months of operation. Collectively, the authors of this study developed a semi-structured interview guide that assessed various domains, including demographics, drug use and overdose history, drug use treatment history, current drug use and recent overdose events, experiences with the CC, purchase frequency at the CC, opinions on drug quality, other CC features, and vision of the CC’s future. We explored processes and early outcomes of the CC via qualitative enquiry among participants; our method allowed for the generation of rich narrative data with potential to reveal several key early findings [18]. This study and all study materials were approved by the University of British Columbia Research Ethics Board (H22-02938).

At the time of interviewing there were 42 members of the CC, all of whom are over the age of 19. The only criteria for inclusion was CC membership for at least 3 months prior to the interview, resulting in 21 eligible members, all of whom were contacted. A prerequisite for membership in the CC was being part of a collective led by people who use drugs, which included (1) BC Association of People on Opiate Maintenance (BCAPOM); (2) The Coalition of Peers Dismantling the Drug War (CPDDW); (3) The Tenant Overdose Response Organizers (TORO); (4) Vancouver Area Network of Drug Users (VANDU); (5) Western Aboriginal Harm Reduction Society (WAHRS). A sample of 16 members participated in an in-depth interview (*n = 16*). Interviews began December 12, 2022 and ended January 13, 2023. To control for potential conflict of interest, authors EN and JK, i.e., DULF founders and operators, were limited to raising awareness about the evaluation among members. They were not told who participated. JB and TK facilitated interviews in private offices separate from the CC site. Prior to interviews, participants reviewed the informed consent document and were offered a hardcopy. Interviewers assessed the capacity of participants at the time of interview to ensure consent was given freely and without coercion. Verbal consent was confirmed by the interviewer. Each participant was remunerated with $40 cash. No participants left the interview early. Participants were queried about the domains listed above. Sample questions asked included, “*Can you tell me a bit about how access to the DULF club has impacted your life(*?*)*,” and “*What changes could be made that would lead you to use the DULF Club more often(*?).” The semi-structured nature of the guide allowed interviewers to then probe according to content stated during the interview.

Interviews were audio recorded and sent to a professional transcriptionist for de-identified transcription (e.g., any names or potentially-identifying information were redacted by the transcriptionist before being sent back to the team for analysis). Following receipt, transcripts were uploaded into NVivo qualitative software [19] for coding and organization. The research team met as a group to discuss the initial coding schema, which included broad categories such as “*purchasing changes*” and “*visiting frequency*.” Our analytic process then explored nuanced emergent themes through a sequential qualitative approach [20] using content and thematic analysis, which included in depth exploration and familiarization of transcripts and a variety of queries conducted by NVivo’s software, which included “*pain*,” “*overdose*,” and “*compassion*.” Field notes and memos were collected to expand upon the relationship between CC experiences, processes, and outcomes. Our deductive reasoning incorporated a priori assumptions, including previous treatment experiences, while our inductive reasoning guided our field observations, helping us infer patterns and uncover unanticipated insights, such as the strong sense of social bonds. Each author has spent prolonged periods in highly saturated drug-using communities, a crucial factor in confirming the validity of our findings [21]. The interviewers debriefed following interviews to discuss early patterns and engage in reflexive processes, and all authors engaged in in-person rigorous inter-author interrogation of the analysis that led to the present manuscript.

### Compassion club operations

**Fig 1:**
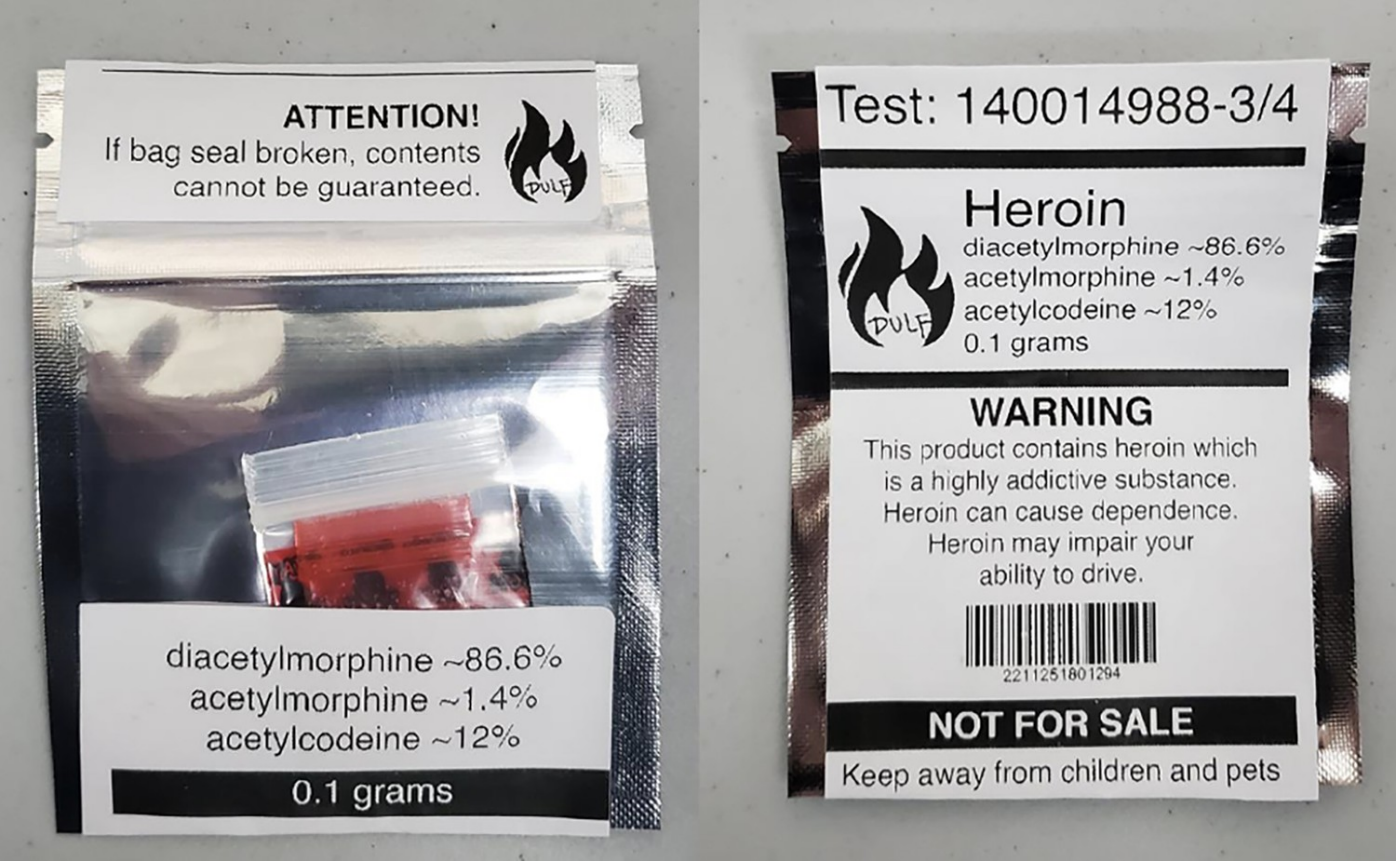
Example of heroin packaging. Permissions Obtained: Eris Nyx, DULF. Drug consumption supplies, naloxone kits, and other resources were available onsite for free. The CC also hosted an overdose prevention site (OPS) where CC members could consume drugs sourced from anywhere (i.e., not just CC-purchased drugs), which kept the same hours as the CC. In addition to the present qualitative investigation, CC members could also participate in quantitative surveys that were collected every three months.

At the time of interview, the CC was open two days per week for 7.5 hours shifts each, and one additional day per week for 3.5 hours. Participants were chosen by lottery, which began with 21 participants who could access the club. The CC ran through a point-of-sale operating system that tracked purchases. Drugs available for sale included heroin, cocaine, and methamphetamine with a known potency and content, and of the highest purity possible, and at a fair cost. Forthcoming research by authors EN & JK assesses and shows the lower cost of CC drugs compared to the unregulated market. Members could purchase up to 7 grams per day or 14 grams per week of each drug. Upon entering the club, members approached a till where volunteer staff took their order and provide the requested drugs. All drugs underwent mass-spectrometry, nuclear magnetic resonance spectrometry, high-performance liquid chromatography, and FTIR Spectrometry and Immunoassay testing prior to being packaged and sold. All packages were sealed and labelled with the contents and a number assigned that corresponded to spectrometry testing results, which could be accessed online (Fig 1).

## Results

### Participant demographics and treatment history

Demographic questions were asked of each participant (*n = 16*) and are detailed in Table 1.

**Table 1:** Sample demographics.

**Gender**	
Woman	6
Man	8
Non-Binary	2
**Average Age**	**48.21**
Race/Ethnicity*	
Indigenous	9
White	10
Other	3
**Sex Orientation**	
Straight	12
LGBTQIA2S+	4
**Housing**	
Private Rental	6
SRO Public	6
SRO Private	3
Homeless	1
**Health/Disability**	
Mobility	5
Heart Disease	1
Chronic Pain	2
HIV	1
Infection	1
Hypertension	1
Mental illness	2
COPD	2
**Drugs recent**	
Fentanyl	6
Heroin	3
Other opioids	5
Cocaine	12
Crack	6
Meth	9
Alcohol	7
Ketamine	3
Benzo	2
DMT	2
Cannabis	8
MDMA	1
**Income**	
FTE	6
PTE	8
Research Stipends	7
Social Assistance	7
Drug selling	2
Other stipends	2
Disability	1
Pension	1
Barter	1

*Acronyms Listed on Acronym & Vernacular List (Page 24)

At the time of interview, nine participants lived in single room occupancies (SROs) and 13 indicated having health conditions that impaired their mobility or respiratory function, along with chronic pain. The average age of participants was 48.2 years (range: 28-to-61); eight identified as men, six identified as women, and two identified as non-binary. Most participants were housed in SROs but had previous experiences of homelessness. Ten participants identified as white, nine identified as Indigenous, and three identified as “other.”

Participants had extensive drug treatment histories, which included inpatient rehabilitation stays, opioid agonist therapies (OATs), and prescriber-run safer drug supply programs. Despite some participants’ histories including time accrued abstinent from drugs, or finding OAT or safer drug supply programs helpful, no participants reported that these programs eliminated their use of the unregulated drug supply entirely for various reasons such as an exceptionally powerful opioid tolerance developed in response to fentanyl, e.g., “*I mean*, *I still take my Kadian (slow-release oral morphine) and my Dilaudids (hydromorphone) every day*, *because it’s a few shovels full on top of the huge mountain of opioid tolerance*. *So*, *I imagine I would probably feel shitty if I didn’t take them”* (P, 6).

### Overdose histories & CC impact

History of overdose was common among participants and was connected to participants descriptions of heavy patterns of use, such as one participant who said their drug use was “*kind of off the charts*” (P, 2). The unpredictability of the unregulated drug supply was the common thread noted among participants to be the root of overdose events. Overdose rates were further aggravated by fentanyl use, as explained by a participant who stated: “*I was ODing (overdosing) quite a bit*. *Like the last time I think was really bad*. *They had to Narcan me like six times*” (P, 4) while another participant stated using drugs from the unregulated supply was a “*gamble with one dice*, *do I live or do I die every time I use*” (P, 9).

Despite reports of overdose histories, participants unequivocally stated that no overdoses resulted from drugs procured at the CC, e.g., “*nobody is overdosing on these (drugs)*” (P, 15). In fact, CC membership itself was said to have an impact on overdose, as noted by one member: “*I haven’t [overdosed] since [being a member]*” and when probed stated that, “*you don’t hear anybody ODing from it at all*, *which is good*, *that’s what we wanted*. *Like that’s what they [CC founders] want to have done*, *is just to have that [overdoses] stop*” (P, 4). Another participant stated that despite their drug use consumption unwavering since joining the CC, they felt safer as “*I don’t have to worry about it (CC drug) being buffed with fent*” (P, 8).

### Purchasing experiences and drug quality

When asked what purchasing drugs was like in a brick-and-mortar store-like space, one participant remarked, “*I think that has been revolutionary*” and “*the (drug) quality and the pricing is topnotch”* (P, 6). Overall, participants trusted that DULF could enhance their safety by acting as a trustworthy regulatory body that provided stable drugs in place of the unpredictable illicit drug market, which is supported by statements such as, “*I really benefit from their excellent quality and pureness which I’ve never really had*. *I’m really really grateful for that … the quality is awesome*” (P, 9) and “*the crystal meth is extremely clean*” (P, 3). Another participant added that adulterants that were associated with overdoses were offset by CC-sourced drugs: *“there is no benzos*, *there is none of the other garbage that you can get on the street”* (P, 15). Another participant contrasted DULF drugs with drugs acquired on the street by rating them as:

*Night and day, I’d say the drugs I get (outside DULF) are about a 6.8 out of 10 and they’re (DULF) a 9.8 … And that’s cause I, you know, and that’s actually pretty high for the drugs I can get with this, like I said I only buy it from two sources. But I can’t give them much more than, 6.8 might be generous out of 10. But DULF is 9.8. So, you really, you know, you can’t get any better. And it’s safe and it’s clean* (P, 9).

Despite the quality of the CC’s drugs, participants did not access the club each day it was open; one participant noted “*Um*, *I’d say I make it there twice a week*” (P, 12). This problem was compounded by financial access and solvency which influenced CC visiting frequency: “*unfortunately I have to (buy on street) because of the hours and the time when I have money sometimes doesn’t coincide with their hours*” (P, 9). When street drugs were purchased, the quality was inconsistent, which was suggested by one participant when asked to describe why CC drugs were preferred: “*for me mostly it’s just about the consistency*, *and the financial aspect*, *and the reliability (of CC-purchased drugs)*” (P, 9). When asked what the experience of access to stable potency drugs was like, another participant furthered the positive outlook by wishing it was available nationally, and hoped the government would see these benefits and be supportive in turn:

*Oh, it’s wonderful, it really is. It is a good thing and I believe that we should have that for the country, not just like this is a small group of people, it’s a research thing as you are well aware of, and it’s a good thing. If we can get the governments to really understand this, that it does help people in day to day living and go and get a job too. Like it doesn’t change me … like I don’t get high off it right, so people know, I’m never nodding or anything like that* (P, 15).

Finally, although uncommon among participants, some strictly purchased drugs from the CC, e.g., “*I only buy from DULF*. *I refuse to use street drugs now*. *I absolutely refuse to*.*”* (P, 8).

### Compassion operationalized and collective decision making

Staff were described as trustworthy and helpful; and gratitude was often expressed for the manner they treated participants, for example, “*[The CC] is accommodating*. *I totally get along great with all the staff*, *like very personable people*, *very respectful*, *you know*, *I have built friendships with them*” (P, 8) and “*It’s a space (the CC) I’m really comfortable in*. *I like the staff*. *They have all been really wonderful*” (P 6). Trust was bolstered by regular communication, including boundaries set and upheld by staff, e.g., as lightheartedly explained by one participant, *“[name redacted] is like*, *‘don’t be calling me at ten after fucking seven (after CC is closed)*, *blah-blah-blah*,’ *(participant laughs)”* (P, 5). Communication also aided in feeling part of a productive community,

*So, I just kept in contact with them (staff), and, you know, they’re constantly, like, throwing me bones, things to do, or, you know, trying to get me involved a little more because they know I’m active in whatever I can be active in. And they just seem to have a little more access to the things going on, so just, I mean …I try to be a part of it* (P, 12).

Regular club meetings integrated a collective decision-making approach for the CC. One participant described how formative implementation meetings operated, “*we had a biweekly meeting where everybody in the study group got together and talked about things*, *and the hours changing was one of the topics*” (P 6). Another participant described how a sense of community resulted from being part of the decision-making processes: *“(it was*) *kind of nice*, *it’s kind of like a bonding thing …you’re looking after each other*” (P, 4). This sense of community described by participants was noted in field notes as a core feature of club meetings. At times, discussions of community led to unprompted descriptions of what “*compassion*” meant with respect to the CC. For some, compassion was the act of opening a CC: “*I like the fact that they (CC founders) care enough to want to do something like this*. *Everybody appreciates that*, *the hard work they do*,” (P6) and,

*I feel trusted and I can trust their evaluation of whatever they’re supplying me with … that’s where I guess the compassion fits in, the where you’re using compassion. Like that’s where it fits, it’s knowing the people and knowing the people makes it trustworthy. If you didn’t know the people, you’re not going to trust them* (P, 8).

This suggests that trust and compassion were developed in multiple ways including biweekly meetings in which participants provided voice that was honored, the observed work ethic of the founders, and, the act of supplying participants with stable drugs that suggested value of participants’ lives. Another participant furthered how compassion was an extension of the social aspects of the CC, and that despite identifying as “*loners*”, they felt cared for by club members:

*The meetings are awesome because I get to, uh, I get to experience what it’s like for people to really care man. You know, and so when I leave you know it just feels like a, like almost family like to founders and other people part of the program actually care about me and everybody else which, which is awesome, you know. And that’s why I have so much respect and I’m so grateful for the program (P, 9).*

The phenomenon of feeling cared for by the CC community appeared to offset the harms of isolation for this participant.

### CC access and participant safety

Participants were queried about their experiences purchasing drugs at the CC in comparison to the unregulated market. One participant commented that when purchasing drugs from the CC, “*I just feel like I’m less open to exploitation or abuse*. *I just feel like the transactional nature of it is just you go in*, *you buy a thing*, *it’s done”* (P, 6). The same participant expanded that the opportunity to purchase drugs without concern of assault resulted in the CC feeling like a store rather than “*copping*”:

*It’s just like you go to a store, and (founder) is there, she’s dressed like a dork, she tells a bad joke, and it’s just like, “Here is your drugs,” and it’s like there’s no mystery, there’s no mystique, there’s no–you know, it’s just like doing any other like transactional sort of thing, like going shopping* (P, 6).

Other participants agreed: “*I like just going to that little store and safe and sound*. *Hi guys*, *you know*, *and they’re nice and friendly and responsible*, *you know*, *because they’re working* (P, 2).” The development of relationships between founders and participants is noteworthy as these relationships can be transformative, as described by a participant,

*I get to go in a nice clean safe environment; very safe. And, and really polite people at the counter and willing to exchange any type of information whether it could be even if I wanted a treatment center, I’m sure they’d give me lots of information too. Any questions about the quality of what I’m buying and it’s just a wonderful, sanitary, and healthy experience as far as I’m concerned* (P, 9).

Staff willingness to facilitate treatment or other resources further exemplified the trust established through the CC’s operations. These positive interactions were also noted in field notes when observing operations. For example, our field notes indicated that participants seemed at ease when on premises, drug use was atypically observed as hasty or rushed, and participants would leave their belongings on a drug consumption table while using the washroom without appearing worried for their items being stolen.

However, positive experiences purchasing drugs at the CC led to some concern about how to go about selecting more members for the CC in the future and worried participants about the potential for violence at the CC:

*There would have to be a screening process. People would have to apply, and be screened. Because if there’s somebody who is well known to be violent–and like I hate excluding people like that, but we need to consider the safety of the staff, and the safety of the other people accessing the resource. Otherwise, you know, you’d be going in there, buying your stuff, and end up in the middle of something really ugly, or the staff who are just working themselves to the bone to provide a really well needed service are putting themselves in danger, and I don’t want that* (P, 6).

Such safety concerns were an extension of the protectiveness and care participants felt for CC founders/staff and other members, in one participant’s words: “*when you’re on a personable basis with somebody (CC staff and participants)*, *you feel safe and safety is key*” (P, 8). While the safety of a brick-and-mortar space was noted for reducing violence, the trusting relationships built between CC founders/staff and participants ultimately bolstered feelings of safety.

### Other health impacts

Overdose risk reduction was the primary aim of DULF’s CC. Nevertheless, participants commented on other health improvements associated with access to the CC. In one instance, while discussing overdose, a participant highlighted the importance of not overindulging on CC drugs despite known composition, “*Like I said*, *doesn’t mean I’m going to be overdoing it and using a whole bunch or anything like that” (P 9)*, suggesting that for this participant, compensatory risk behavior was not a response to access to, and use of, unadulterated drugs. Benefits described not only included enhanced personal safety but extended to the safety of other CC members:

*I do, I appreciate the fact that it’s (CC) there. I’m so grateful. I’m very very grateful because honestly, I think DULF saves lives. Like from what I’ve seen … there’s a bunch of people that I know would have died like months ago. If they hadn’t been on CC because they would have been grabbing whatever* (P, 8).

When queried about health, responses included improvements, including health improving *“tremendously*, *I feel so much better*” (P, 16) and belief of future health benefits “*my health is going to improve”* (P, 9).

Pain relief was an important health outcome that was associated with access to the CC. In one example, a participant was able to control their pain with heroin: “*A little bit of (cocaine)*, *yeah*, *just some energy and then some opioids for the pain*” (P, 3), while another participant mentioned how CC-sourced drugs helped their chronic pain “*all the time*, *yeah*” (P, 8). Another participant with chronic pain furthered that not relying on medical providers to treat their pain was a bonus as there was no denigrating oversight involved: *“I probably could go to my doctor (for pain)*, *but I’d just as soon not*. *I don’t care for doing piss tests and having him follow me around in that respect”* (P, 15). Ability to control pain autonomously by finding the right doses of drugs needed in real time, as opposed to waiting for a healthcare provider to adjust medications or doses, was indicative of difficulties having pain adequately addressed through the medical system.

For participants who primarily used stimulants, access to uncontaminated drugs similarly functioned to reduce stress of consuming substances of unknown purity and composition that could lead to undesired outcomes and other health issues. For example, contaminated cocaine was identified as leading to adverse health outcomes that were starting to resolve through access to unadulterated CC-sourced cocaine: “*It’s about my health*, *my health and I’ve been suffering from side effects (of levamisole) for decades and … I’d notice some of my poisoning symptoms actually do not disappear but totally*, *got way reduced (from DULF cocaine)*” (P 9) and furthered “*that what they’re doing is absolute harm reduction 101 … a lot of my symptoms from levamisole poisoning are almost gone*.”

### Titrating onto heroin

Consuming heroin from the CC was complicated for participants that had an established tolerance for fentanyl, as heroin is a considerably less potent opioid, e.g., “*And then when I find heroin*, *because fentanyl’s so strong*, *it wouldn’t do anything*” (P, 12). Transitioning from illicitly-manufactured fentanyl to CC-sourced heroin was a goal expressed by many participants but was accompanied by noted titration challenges, for example, “*the transition back to heroin*, *I mean*, *that’s the thing*, *it’s not like it can go from fentanyl to heroin overnight*,” but simultaneously attempted tactics to try: “*I’ve been buying heroin (at CC) and mixing it in with my fentanyl to try and go down on my fentanyl and up on the heroin*” (P, 12). Transitioning to heroin use after developing a fentanyl tolerance was further complicated other substances like benzodiazepines in illicitly-manufactured fentanyl, which created additional physiological dependencies and subsequent withdrawal symptoms that heroin alone could not assuage. This was supported by comments such as:

*Like buying down (fentanyl) off the street now, so much of it has benzos in it, and benzos are so hard on a body, and once you get physically dependent on those, they’re harder to quit than the down, if you were to choose to quit, but just psychologically it fucks with your head too* (P, 15).

The CC’s uncontaminated heroin was nonetheless viewed as a safety approach, “*you know*, *it’ll eliminate*, *hopefully*, *a lot of the safety concerns of getting benzo-dope or getting stuff that you don’t want”* (P, 6).

### Suggested improvements

Despite overall positive feedback, participants were asked if they had any suggested improvements for the CC. Responses included the aforementioned desire for uncontaminated fentanyl to be available for purchase: “*The only complaint that I have is that they don’t sell fent*, *and the hours aren’t*, *you know*, *open enough for me*” (P, 6) and that participants asked in *“every meeting we have*, *‘Can we get fentanyl*?*”* (P, 1). Another participant expanded on wishing the CC was open for longer hours and more days per week, “*I don’t get in to pick up my dope because it’s just it’s only open for a few hours a couple days a week*, *and it always ends up being during one of my (work) shifts”* and continued that *“I really need them to alter their hours there*. *I need it like*, *all the time*” (P, 12). Another participant had trouble keeping track of the days and hours of CC operation, e.g., “*we need to have signs out there that says what time they’re open …some people don’t know … like me*” (P, 11). Others suggested expanding the club to more members, “*opening it up to more people*, *without compromising safety”* (P, 6) and “*opening up to more people is gonna help a lot*” (P, 8). Another participant expressed desire for work opportunities as the CC: “*It’d be nice to see more employment opportunities for the folks to be involved*, *because we all want to be involved*, *and we all have different skillsets*” (P, 6). A few participants mentioned that a delivery service would be helpful, especially for those with mobility issues, e.g., “*what would be nice is like if they can deliver if you’re close by*” (P, 4). Despite these suggestions, some participants had no suggested improvements, “*I don’t see how*. *We don’t*, *you know that saying don’t fix what ain’t broke*” (P, 8) and “*But I think for what it is*, *and for the scale and the size and the staffing*, *it’s pretty much as good as it can be*” (P, 6).

## Discussion

The present evaluation revealed a range of themes and outcomes expressed by participants related to health and social benefits associated with engagement with DULF’s compassion club (CC). Despite the fact that the majority of participants had previous engagement with the “addiction medicine” system, none whom were queried found such interventions adequate. Although DULF’s model suffered from some limitations primarily due to access restrictions and a lack of drug selection, no participants reported experiencing or observing any overdoses associated with CC-sourced drugs. This suggests the CC cultivated an environment in which harm reduction practices were being employed by members. The autonomy provided by the CC to purchase drugs of predictable content without medical oversight might have enhanced CC members’ ability to reduce harm and increase their agency and sense of belonging.

To that end, the social bonds created with other CC members, founders, and staff facilitated feelings of safety, respect, and trust; which is especially important amongst stigmatized groups such as people who use drugs as they are less likely to feel respected, validated, or receive life-affirming treatment in many institutions meant to support their health and safety [22–25]. Feelings of belonging were facilitated by compassionate social bonding developed via inclusive horizontal decision-making, and risk stratification by club founders, which was deemed an act of care. Members also noted early CC-related health improvements, such as addressing chronic pain, which shows promise that added positive health outcomes might continue, but can only be determined with time and further investigation.

The present study also reconfirmed that, for some, buying drugs from the illicit market was accompanied by risk of exploitation and abuse. Experiences and concerns of violence when consuming drugs in public or purchasing drugs from the illicit market are drivers of using drugs alone, which is a key risk factor for overdose death [26–28]. However, the CC revealed to be social and physical space in which personal safety was cultivated and may have offset risks commonly experienced in the context of the Downtown Eastside [29, 30], subsequently providing a refuge from worry of violence or exploitation. Even self-described “loners,” who mentioned preferring using drugs alone, noted enjoying the social bonds that were developed at the CC, which was an unexpected benefit. Seeing as no CC-sourced drugs were implicated in any overdose events and that the CC developed an important consumer safety measure against systemic violence, the CC was especially important for people who purchase and use drugs alone.

Although the potency of fentanyl has resulted in an exceptionally powerful opioid tolerance, and the unregulated market has possibly created additional physiological dependencies on other substances like benzodiazepines that can lead to seizures if abruptly stopped [31], opioid-using CC members in the present study expressed desire and described attempts to transition to using stable heroin sold at the CC. This effort suggests that some people who use drugs are trying to steer away from the fentanyl-saturated illicit opioid market in favor of community-regulated heroin. Lessons from clinical titrations from fentanyl to methadone, and benzodiazepine tapers for those interested, might be helpful [32, 33]. This could further be assisted through additional community support for the fentanyl-to-heroin transition or by the CC being legally authorized to introduce regulated fentanyl and benzodiazepine products into their operations.

Despite the CC’s positive impacts on its membership, such projects have received little uptake despite the medical system and treatment sector’s inefficiencies. Sufficient capacity-to-scale is a noted barrier to safe drug supply programs implemented through medical prescribing [13]. Despite several positive outcomes associated with safer supply drug programs—such as reductions in overdose risk, illicit drug use, number of daily injections, and healthcare costs [34–39]–these programs might not be available to or preferred by all people at risk of overdose. Barriers to safe supply programs include an already overburdened health system in Vancouver constraining prescribing to meet need and a subset of prescribers having expressed discomfort bearing the responsibility of safe supply provision [13, 38, 39]. Some people who use drugs are also disinterested in any intervention that connects their drug use to the healthcare system [14]. The CC, however, was unconnected to healthcare systems, described as being “like a store,” and provided autonomy and anonymity in drug purchasing that prevented members from subjecting their drug use to compulsory medical surveillance. In lieu of drug prohibition’s end and the development of a fully regulated and non-medicalized drug market, the CC model has demonstrated overdose prevention and beneficial health outcomes. To rapidly scale-up the CC model and expand access to drugs with predictable content to people otherwise at risk of harms from the unregulated drug supply, the CC’s low-barrier, non-medicalized, and drug-user led approach is fertile ground for future investigation.

### Limitations

Our study is not without limitations. Qualitative investigation is often subject to social desirability bias [40]. Interviewers made efforts to reduce the potential for this bias by holding interviews offsite and ensuring participants that their identity would not be disclosed to CC founders and they could speak freely about the intervention without worry of reprisal. Interviewer bias is a potential consideration due to our preconceived support for drug compassion clubs. However, in qualitative research, acknowledging such beliefs upfront differs from bias [41]. This methodology, grounded in constructivism, recognizes that interviewer perspectives may influence data collection. Importantly, both data collectors bring decades of experience in qualitative research, regularly engaging in reflexive practices to address any influence when necessary. The small sample size of our study also limits the transferability of the findings [42], however small sample sizes are the hallmark of qualitative data and our semi-structured interview approach allowed probing of unexpected emergent findings, such as the social bonds expressed by many participants in the present study.

## Conclusion

Given the fact that results from this preliminary study seem favourable, the CC model seems fertile grounds for future innovation. This stems from participants statements regarding its ability to have: prevented overdoses and ensured drug quality; helped develop positive consumption habits; created a compassionate community with horizontal decision-making but stratified risk; and led to better health outcomes. No participant acknowledged overdose events stemming from CC-sourced drugs indicating early beneficial outcomes associated with DULF’s CC. This suggests that access to and use of drugs with a predictable and known content, combined with a trusted environment that promoted social bonds, might have even greater effect if expanded. The CC approach of selling uncontaminated and tested drugs in a safe environment is an important innovation in the spectrum of overdose prevention interventions and ought to be legally supported and evaluated, especially as the contaminated illicit drug supply shows no indication of stabilization or reverting to predictability.

## List of abbreviations

BCAPOM: *BC Association of People on Opiate Maintenance*
Benzo: Benzodiazepine
Buffed: Adulterated
CC: Compassion Club
CPDDW: The Coalition of Peers Dismantling the Drug War
COPD: Chronic Obstructive Pulmonary Disease
Dilly/Dilllies: Hydromorphone (Brand name: Dilaudid)
DMT: Dimethyltryptamine
Dope: Fentanyl
Down: Fentanyl
DTES: Downtown East Side
DULF: Drug User Liberation Front
Fent: Fentanyl
FTE: Full time employment
Meth: Methamphetamine
PTE: Part time employment
SRO: Single residence occupancy
OAT: Opioid agonist therapy
OD: Overdose
OPS: Overdose prevention site
SROM: Slow-release oral morphine (e.g., “Kadian”)
TORO: The Tenant Overdose Response Organizers
WAHRS: Western Aboriginal Harm Reduction Society
